# Surface Passivation with a Perfluoroalkane Brush Improves the Precision of Single-Molecule Measurements

**DOI:** 10.1021/acsami.2c16647

**Published:** 2022-10-28

**Authors:** Carlos J. Bueno-Alejo, Marina Santana Vega, Amanda K. Chaplin, Chloe Farrow, Alexander Axer, Glenn A. Burley, Cyril Dominguez, Hesna Kara, Vasileios Paschalis, Sumera Tubasum, Ian C. Eperon, Alasdair W. Clark, Andrew J. Hudson

**Affiliations:** † School of Chemistry, University of Leicester, University Road, Leicester LE1 7RH, United Kingdom; ‡ Leicester Institute of Structural & Chemical Biology, Henry Wellcome Building, University of Leicester, Lancaster Road, Leicester LE1 7HB, United Kingdom; § School of Engineering, Advanced Research Centre, University of Glasgow, 11 Chapel Lane, Glasgow G11 6EW, United Kingdom; ∥ Department of Molecular and Cellular Biology, Henry Wellcome Building, University of Leicester, Lancaster Road, Leicester LE1 7HB, United Kingdom; ⊥ Strathclyde Centre for Molecular Bioscience & Department of Pure & Applied Chemistry, University of Strathclyde, 295 Cathedral Street, Glasgow G1 1XL, United Kingdom

**Keywords:** single-molecule approaches, interferometric scattering microscopy, mass photometry, surface passivation, polymer brushes, nanofabrication, protein complexes

## Abstract

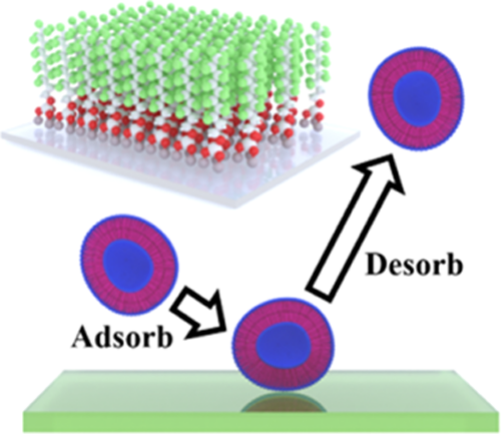

Single-molecule imaging is invaluable for investigating the heterogeneous behavior and interactions of biological molecules. However, an impediment to precise sampling of single molecules is the irreversible adsorption of components onto the surfaces of cover glasses. This causes continuous changes in the concentrations of different molecules dissolved or suspended in the aqueous phase from the moment a sample is dispensed, which will shift, over time, the position of chemical equilibria between monomeric and multimeric components. Interferometric scattering microscopy (iSCAT) is a technique in the single-molecule toolkit that has the capability to detect unlabeled proteins and protein complexes both as they adsorb onto and desorb from a glass surface. Here, we examine the reversible and irreversible interactions between a number of different proteins and glass *via* analysis of the adsorption and desorption of protein at the single-molecule level. Furthermore, we present a method for surface passivation that virtually eliminates irreversible adsorption while still ensuring the residence time of molecules on surfaces is sufficient for detection of adsorption by iSCAT. By grafting high-density perfluoroalkane brushes on cover-glass surfaces, we observe approximately equal numbers of adsorption and desorption events for proteins at the measurement surface (±1%). The fluorous–aqueous interface also prevents the kinetic trapping of protein complexes and assists in establishing a thermodynamic equilibrium between monomeric and multimeric components. This surface passivation approach is valuable for *in vitro* single-molecule experiments using iSCAT microscopy because it allows for continuous monitoring of adsorption and desorption of protein without either a decline in detection events or a change in sample composition due to the irreversible binding of protein to surfaces.

## Introduction

Single-molecule approaches can be used to unravel the complexity of protein behavior and reveal information that is concealed by ensemble measurements.^1−4^ By far, the most common experimental methods utilize single-molecule fluorescence, where either freely diffusing biomolecules are probed individually by spatial filtering using confocal microscopy (*via* fluorescence resonance-energy transfer,^5^ correlation spectroscopy,^6^ or alternating laser excitation spectroscopy^7^), or surface-tethered biomolecules are illuminated by an evanescent field using total internal reflection fluorescence microscopy (TIRFM).^8^ These methods require the labeling of molecules with a fluorescent probe. Label-free experimental approaches are beginning to be adopted more widely, such as interferometric scattering microscopy (iSCAT),^9^ which detects individual molecules for a transient moment in time as they are in the process of adsorbing onto or desorbing from a microscope cover glass. iSCAT has recently been optimized for mass detection of proteins, and this application of the technique is referred to as mass photometry.^10,11^ By eliminating the requirement to fluorescently label components, the experiments will not be adversely affected by hydrophobic interactions mediated by the addition of a fluorescent tag.^12,13^

In common with all variants of single-molecule microscopy, iSCAT is adversely affected by irreversible adsorption of biomolecules onto cover glasses. The irreversible adsorption of protein prevents the detection of desorption events by iSCAT microscopy, which is useful information to facilitate the determination of the masses of different proteins in a sample. It can also change the heterogeneous constitution of the sample due to differences in the affinity between various components and the glass surface. It will cause the displacement of a dynamic equilibrium between protein multimers and monomers as the total concentration in solution is lowered.

The cleaning of cover glasses for single-molecule experiments creates terminal silanol groups on the surface, which deprotonate in aqueous environments, leaving a negative interfacial charge density. There are a number of different methods designed to minimize adsorption of biomolecules to negatively charged glass surfaces.^14−16^ The most common is the passivation of surfaces by either unlabeled proteins or synthetic polymers. In protein blocking methods, the binding sites on a glass surface are populated by an inert protein and become unavailable to make interactions with other molecules.^17−20^ The most common protein used for this purpose is bovine serum albumin (BSA), but the method suffers from the weakness of the noncovalent interaction of BSA with the surface-binding sites, leading to a lack of uniformity of the inert protein layer.^14^ Improved passivation is obtained by grafting a high-density hydrophilic, polymer brush in a monolayer coating on the glass surface. A suitable polymer, such as poly(ethylene glycol) (PEG), can be covalently tethered to the surface using a succinimide-functionalized derivative on a glass surface pretreated by an aminosilane reagent.^21−23^ PEG is a non-ionic water-soluble polymer in which the ethylene glycol groups form hydrogen bonds to water molecules, creating a hydration layer to inhibit surface interactions with proteins.^14,24−27^ The polymer chain is highly flexible, with a short persistence length,^28^ and end-grafted PEG will form a random coil on the surface (“mushroom-like” configuration), which inhibits the formation of a high-density brush, leaving potentially exposed areas of glass.^29−33^ An improved density of polymer has been obtained by the formation of the brush under conditions of marginal solubility (*i.e.*, at high ionic strength). This results in compact coiled structures of end-grafted PEG, which will transition to an equilibrium random coil as the ionic strength is reduced and thereby fill the gaps between the covalently attached molecules.^22^ Nevertheless, there are proteins with a high affinity for PEG, which will always limit its suitability for passivating surfaces in single-molecule experiments.^34−36^ Other researchers have tried alternative strategies to passivate surfaces by the formation of a high-density brush using different functionalized polymers.^37−39^

For iSCAT microscopy, the measurement surfaces cannot be reliably passivated with a coating of PEG due to the coiled structure and conformational mobility of polymer chains, which creates background noise in contrast levels, and substantial variability in different regions of interest on the cover-glass surface, preventing the detection of single molecules. Here, we describe a type of high-density brush formed from perfluoroalkanes, −(CF_2_)*_n_*CF_3_, where *n* ≥ 4, which, in contrast to PEG, creates a hydrophobic interface. Perfluorinated compounds associate with each other to maximize fluorine–fluorine interactions at the expense of any other type of interaction,^40,41^ a phenomenon known as the fluorous effect.^42^ As a consequence of the fluorophilicity of the interface, both water molecules and hydrated protein molecules will be excluded from the interstices in the brushes. Using iSCAT microscopy, we are able to observe the time course of protein interactions at the interface between a perfluoroalkane brush and an aqueous sample and distinguish between reversible and irreversible adsorption of protein.

The ability of perfluorinated compounds to reduce adsorption has been exploited for industrial and everyday consumer products for several years, for example, to fabricate nonstick cooking utensils or as water and grease repellents for carpets and clothing. More recently, perfluoroalkane brushes have been employed as anti-fouling coatings for biomedical applications,^43,44^ and copolymers containing fluorous moieties have been developed for medical diagnostics^45,46^ or biofouling release of marine organisms.^47^ In this work, we present the first example of a perfluorinated monolayer being used for single-molecule imaging. We have demonstrated previously that micropatterning of DNA on a perfluoroalkane brush can be achieved by covalent modification of oligonucleotides with a short perfluoroalkane tag, whereby the fluorous effect is exploited to tether DNA to interfaces by the insertion of its tag into the interstices in the brush.^48^ Others have shown the capability to prepare a protein microarray using fluorous–fluorous interactions.^49^ In the present work, we aim to show that it is possible to eliminate the irreversible binding of protein to a measurement surface and demonstrate the potential of this type of nanopatterning on surfaces for experimental studies using iSCAT microscopy.

## Results and Discussion

### Formation of Perfluoroalkane Brushes on Glass

**Figure 1:**
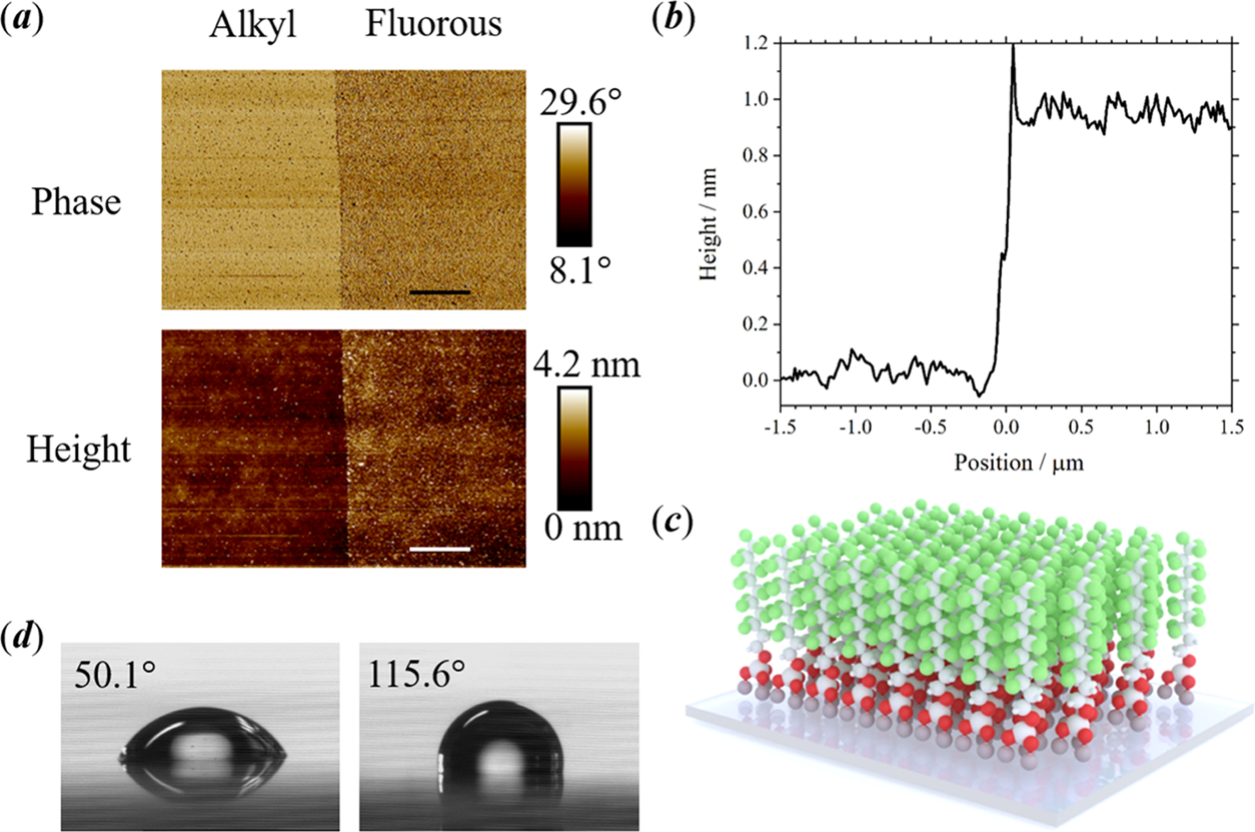
(a) Tapping mode AFM of the interface between SAMs of two different polymers on a glass substrate (borosilicate glass microscope slide). Both polymers comprise a chain length of 10 carbon atoms (left side, hydrocarbon; right side, perfluorinated compound). Individual polymer molecules were covalently bonded to the glass substrate using silane chemistry. Top—the image of the phase difference between actuation and displacement of a silicon-cantilever tip. Bottom—the image of the surface topography. Scale bar represents 1 μm. (b) 256 lines of the AFM image have been averaged to illustrate the vertical step between the hydrocarbon and perfluorinated compound SAMs. The profile was corrected for the tilt of the sample (−0.3 nm in height per 1 μm in horizontal position). (c) Illustration of the SAM of the perfluorinated compound on glass, which leads to the formation of a polymer brush. (d) Contact angle characterization of a 5 μL-sessile droplet of reverse osmosis water on an uncoated (50.1°) glass substrate (ultrasonically cleaned in acetone and isopropyl alcohol) and a fluorous-coated (115.6°) glass substrate.

Self-assembled monolayers (SAMs) of a perfluorinated compound were generated on glass substrates *via* gas-phase deposition of (heptadecafluoro-1,1,2,2-tetrahydrodecyl)triethoxysilane (see Materials and Methods section). The compound binds covalently to the glass substrate through the terminal silane group. To aid characterization of the physical properties of the fluorous coating, a comparison was made between the SAMs formed by the perfluorinated compound and a corresponding hydrocarbon, decyltrimethoxysilane. Adjacent areas of a glass substrate were exposed to the two different silane-functionalized compounds. The procedure used to mask areas of the glass substrate from exposure to one type of reagent is outlined in the Materials and Methods section. The interface between SAMs of the hydrocarbon and the perfluorinated compound was examined by an atomic force microscope (AFM) operating in tapping mode. The phase difference between actuation and displacement of the silicon-cantilever tip is shown in Figure 1a across a surface area of 3.85 by 7.7 μm (256 lines, 512 samples per line; pixel size of 15.0 nm). The two different SAMs are clearly distinguishable in the image, where a smaller phase difference is observed in the fluorous-coated area (right—mean, 21.5°; standard deviation, 3.2°) compared to the alkyl-coated area (left—mean, 23.6°; standard deviation, 1.9°), suggesting that the fluorous coating has a weaker adhesive interaction with the silicon-cantilever tip.

The surface topography is illustrated in the lower image shown in Figure 1a, with a one-dimensional (1D) profile obtained by averaging the height measured in each of the 256 lines of the image shown in Figure 1b. A step size of approximately +0.9 nm is observed between the SAMs of the hydrocarbon (left) and the perfluorinated compound (right). The magnitude of this height difference informs on both the conformational geometry of the perfluorinated compound and the alignment of the carbon chains relative to the surface plane of the glass substrate. An estimate of the length of the fully extended carbon chain for the perfluorinated compound is 1.24 nm, assuming an anti-conformational geometry about each carbon–carbon bond and the terminal carbon–fluorine bond (bond length data from ref (50)). A similar length of 1.22 nm can be estimated for an all-anti-conformational geometry of the corresponding hydrocarbon. The step in height between the alkyl- and fluorous-coated areas of the glass surface is +0.9 nm, and this magnitude can only be explained by the perfluorinated compound favoring an all-anti-conformational geometry (as illustrated in Figure 1c), in contrast to the alkyl compound favoring disordered conformational geometries. The hydrocarbon must be coiled along the 10-carbon chain length, with the terminal methyl groups located ∼0.3 nm (or less) from the glass surface.

The contact angle of a sessile drop (5 μL) on a perfluoroalkane brush grafted onto a glass substrate was measured to be 115.6° compared with a smaller angle of 50.1° on an uncoated glass substrate, which confirms the expected increase in surface hydrophobicity; see Figure 1d.

### Interaction of Proteins with a Perfluoroalkane Brush

**Figure 2:**
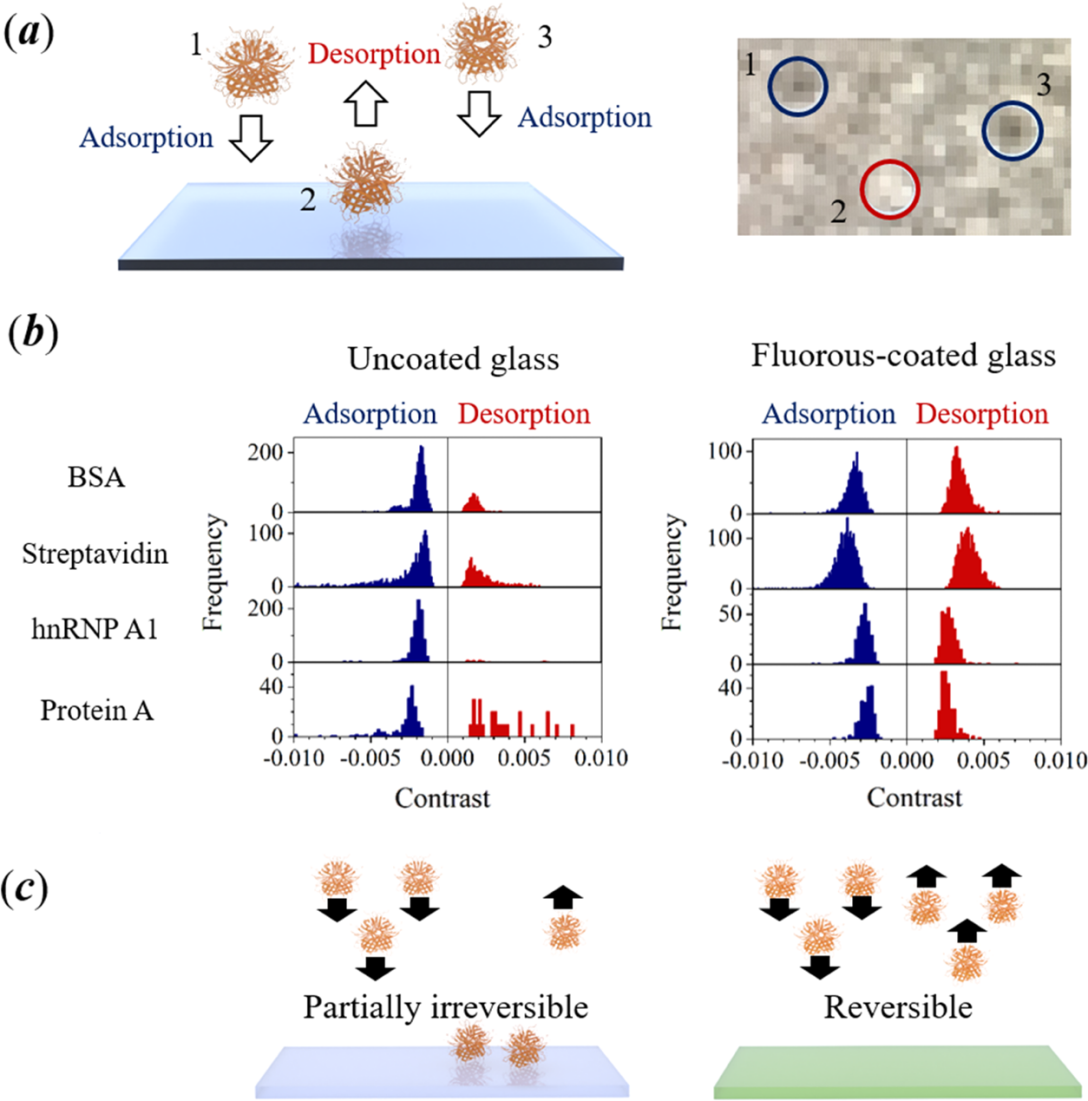
(a) Illustration of the detection of adsorption and desorption of protein at the single-molecule level (cartoon representation on the left) by iSCAT microscopy (video image on the right), where negative contrast (enclosed by blue circles) and positive contrast (enclosed by red circles) are observed for adsorption and desorption of single protein molecules, respectively. (b) Histograms of the frequency for adsorption (blue) and desorption (red) events as a function of contrast in interferometric images. The duration of each experiment was 60 s. Data has been obtained for four different proteins (BSA, streptavidin, hnRNP A1, and protein A) dispensed in a 10 μL volume of a 20 nM solution (in T50 buffer; see Materials and Methods section) onto uncoated cover glasses (left) and fluorous-coated cover glasses (right). The total number of counts for adsorption events, relative to desorption events, are: BSA—1573/518 (uncoated), 995/1003 (fluorous-coated); streptavidin—958/330 (uncoated), 1436/1434 (fluorous-coated); hnRNP A1—810/17 (uncoated), 211/215 (fluorous-coated); protein A—137/9 (uncoated), 199/197 (coated). Small numbers of adsorption and desorption events will not be detected by the instrument. (c) Illustration of an aqueous protein sample in contact with an uncoated (left) and a fluorous-coated (right) glass surface. Irreversible adsorption of the protein occurs on uncoated glass.

The label-free approach, iSCAT microscopy, is ideal to characterize how proteins interact with a perfluoroalkane brush. This measurement technique has recently been adapted to measure the mass of proteins in aqueous samples dispensed onto a cover glass.^10^ In iSCAT microscopy, interferometric images are recorded between the light reflected from the interface between a cover glass and an aqueous sample and elastic-scattered light from proteins adsorbed on the cover-glass surface. By analyzing a change in the interferometric image, from one moment to the next, the presence of a protein is detected at the moment of either adsorption or desorption from the measurement surface; see Figure 2a. Adsorption and desorption of a single protein lead to negative and positive contrast, respectively, in a background-subtracted image from a video recording. The magnitude of the change in contrast is dependent on the molecular weight of the protein. In our experiments, iSCAT has been implemented on an inverted microscope system utilizing a confocal-scanning 520 nm laser. The instrument is a commercially available mass photometer (OneMP) manufactured by Refeyn Ltd. (see Materials and Methods section). The acquisition and analysis of images to determine the adsorption and desorption of single proteins were made by a software package called AcquireMP (Refeyn Ltd.); further analysis of data was performed using software DiscoverMP (Refeyn Ltd.) and OriginPro 2021 (OriginLab).

Here, we have compared the number of adsorption events with the corresponding number of detected desorption events for different proteins to determine the degree of nonspecific binding to measurement surfaces. The surface dynamics were studied for bovine serum albumin (BSA), streptavidin, heterogeneous ribonucleoprotein A1 (hnRNP A1), and protein A on both uncoated glass and a perfluoroalkane brush grafted onto glass. BSA and streptavidin were selected because they are widely used in single-molecule measurements, and BSA is known to readily form a coating on measurement surfaces; hnRNP A1 is a RNA-binding protein and has a high affinity for intermolecular interactions; protein A has a relatively low molecular weight and could have a transient interaction with surfaces, which will make it more challenging to detect the adsorption of the protein by iSCAT. Each of these proteins was studied in separate experiments by dispensing a 10 μL volume of a 20 nM solution (in T50 buffer) onto uncoated and fluorous-coated cover glasses; the total duration of each experiment was 60 s. Histograms of the frequency counts of adsorption and desorption events as a function of contrast in interferometric images are shown in Figure 2b for each of these four proteins. At the low concentration of 20 nM, only a single peak is observed in the frequency distribution for BSA, and there is no apparent dimerization of the protein; hnRNP A1 and protein A are also monomeric proteins. However, streptavidin remains a tetrameric complex at this concentration. For the experiments on uncoated glass (left), the frequency of adsorption events (shown in blue) is significantly higher than that for desorption events (shown in red); see figure caption, *ca*. 3× higher for BSA and streptavidin, 50× for hnRNP A1, and *ca*. 15× for protein A. This indicates that, in the majority of instances, the adsorption of these proteins on uncoated glass is irreversible. In contrast, on fluorous-coated glass (right), the frequency of adsorption and desorption events are similar (within ±1%, see figure caption) and hence the adsorption of these four proteins is reversible. iSCAT measurements are made across a region of interest (ROI) of 3 × 10 μm^2^ on cover-glass surfaces. To demonstrate the homogeneity of the perfluoroalkane brush across a wider area, replicate measurements of the histograms shown in Figure 2b were obtained at four different locations for protein samples of BSA and streptavidin (see Figures S1 and S2). For each separate ROI, similar numbers of adsorption and desorption events were detected.

**Figure 3:**
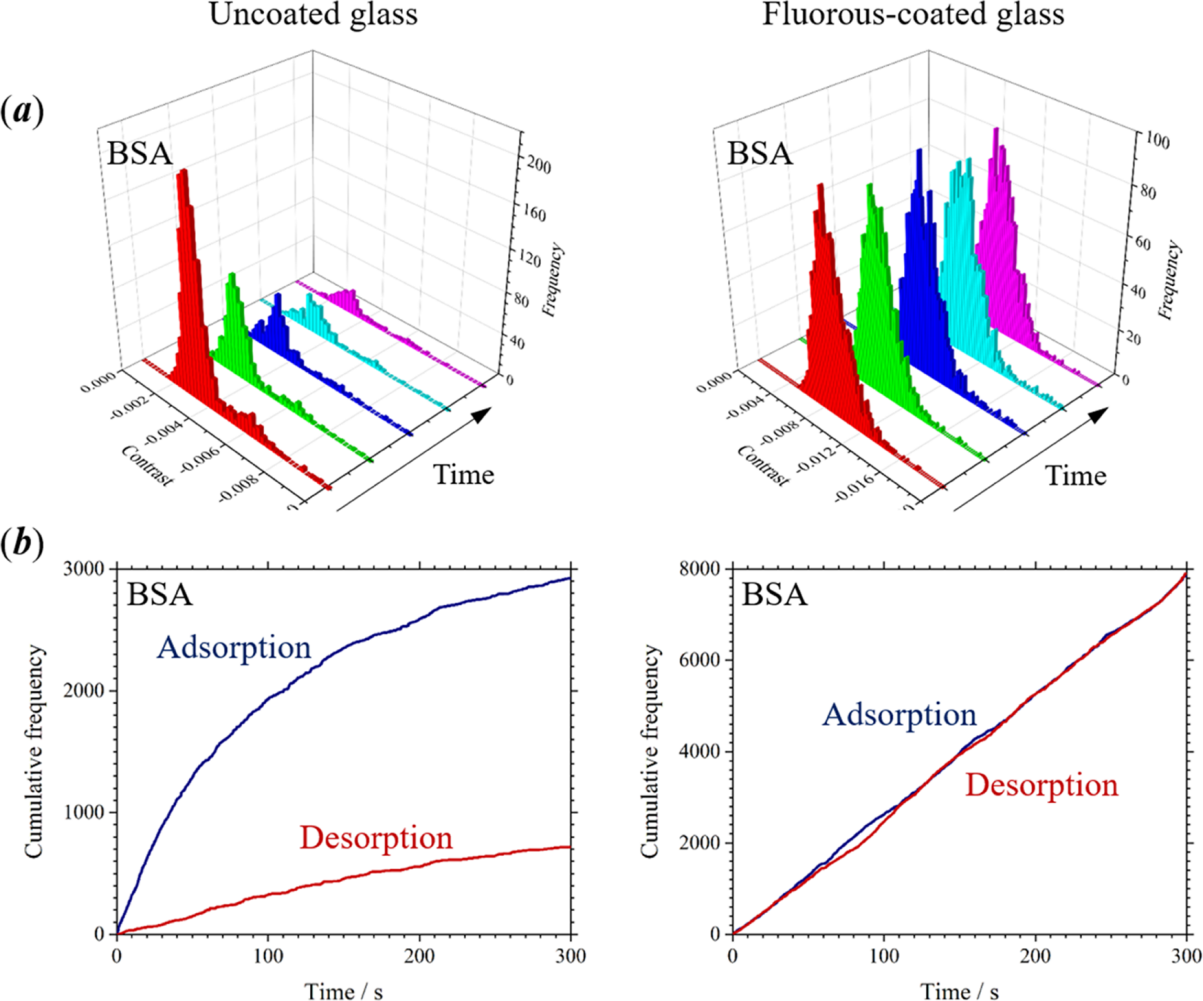
(a) Sequential measurements of the frequency for adsorption of BSA onto uncoated cover glasses (left) and fluorous-coated cover glasses (right). Each of the histograms represents an interval of 60 s (total experiment time was 5 min). Data were obtained after a 10 μL volume of a 20 nM solution of BSA in T50 buffer was dispensed (no further additions of protein were made after the start of the experiment). (b) Time dependence of the cumulative frequency for adsorption (blue) and desorption (red) of BSA on uncoated (left) and fluorous-coated (right) glass. Corresponding data for the time dependence of the cumulative counts for adsorption and desorption of streptavidin, hnRNP A1, and protein A on uncoated and fluorous-coated cover glasses are provided in the Supporting Information.

The concentration of the aqueous protein samples will remain constant when in contact with the fluorous-coated glass, while the concentrations will decline as a consequence of the irreversible adsorption of protein on uncoated glass; see illustration in Figure 2c. The use of fluorous-coated surfaces could ensure a higher precision for single-molecule measurements reliant on maintaining fixed concentrations of components. To demonstrate this, we have measured histograms for the frequency of adsorption of BSA on uncoated and fluorous-coated glass at sequential intervals in time. The data in Figure 3a show a continuous measurement across 5 min, in which the frequency counts are separated into 60 s intervals. As before, a volume of 10 μL of a 20 nM solution of BSA in T50 buffer was dispensed at the start of the experiment on each of the two different surfaces. On uncoated glass, there is a decline in the frequency of adsorption events in successive measurements (to approximately 1/10 of the initial frequency) due to the decreasing concentration of protein in the dispensed sample, whereas, on fluorous-coated slides, the frequency of adsorption events remains constant over the experimental time of 5 min.

The cumulative frequency of adsorption events might, at first, appear to be too low to account for a decrease in protein concentration in the dispensed droplet. For example, a cumulative frequency for adsorption of BSA on glass of just under 3000 was obtained after 5 min (Figure 3b). The field of view in iSCAT images is 30 μm^2^, and the contact area of the aqueous protein samples on the cover glass was 13 mm^2^; hence, the total number of adsorption events over the entire contact area must have been 1.3 × 10^9^. To account for a substantial depletion of protein concentration, which was initially 20 nM in the 20 μL sample volume (corresponding to 2.4 × 10^11^ molecules), the detection efficiency for adsorption events by iSCAT must be below 10%. A simple back-of-the-envelope calculation can be made to obtain a lower limit for the number of collisions of protein molecules with the cover-glass surface (see Supporting Information). We estimate that more than 1.5 × 10^10^ molecules of protein, with a typical diffusion coefficient of 10^–6^ cm^2^ s^–1^, could adsorb onto the cover glass after an interval of 5 min, which implies an upper limit for the detection efficiency of iSCAT microscopy for single-molecule adsorption of 8%.

Plots of the cumulative frequency for both adsorption and desorption of BSA on both uncoated and fluorous-coated glass as a function of time are shown in Figure 3b. On uncoated glass, the temporal profile for the cumulative counts for adsorption and desorption are nonlinear, with a negative curvature, and the number of desorption events is much lower. In contrast, the temporal profile is linear and nearly identical for both adsorption and desorption of BSA on fluorous-coated glass. A plot of the cumulative frequency for adsorption of BSA over a much longer time interval of 17 min is shown in Figure S3. Over this longer interval of time, the plot for the cumulative frequency shows a slight positive curvature due to evaporation of water molecules and hence the concentration of the protein sample leading to increasing frequencies for adsorption and desorption of protein on the surface. Corresponding data for the cumulative frequency for adsorption of streptavidin, hnRNP A1, and protein A as a function of time on uncoated and fluorous-coated cover glasses are provided in the Supporting Information (see Figure S4). The linear plots of cumulative frequency obtained for protein A on the perfluoroalkane surface are especially significant, since protein A is known to bind to components *via* hydrophobic interactions.^51,52^ This result shows that the adsorption of a protein with hydrophobic domains is still reversible on a perfluoroalkane brush.

### Interaction of Multimeric Protein Complexes with a Perfluoroalkane Brush

The previously studied proteins existed as either monomer units (BSA, hnRNP Al, and protein A) or remained as a stable multimer (streptavidin) at the low experimental concentration (20 nM). The results described in this section were obtained from lactate dehydrogenase (LDH), which forms a tetramer, and Ku70/80, which forms a heterodimer complex.

**Figure 4:**
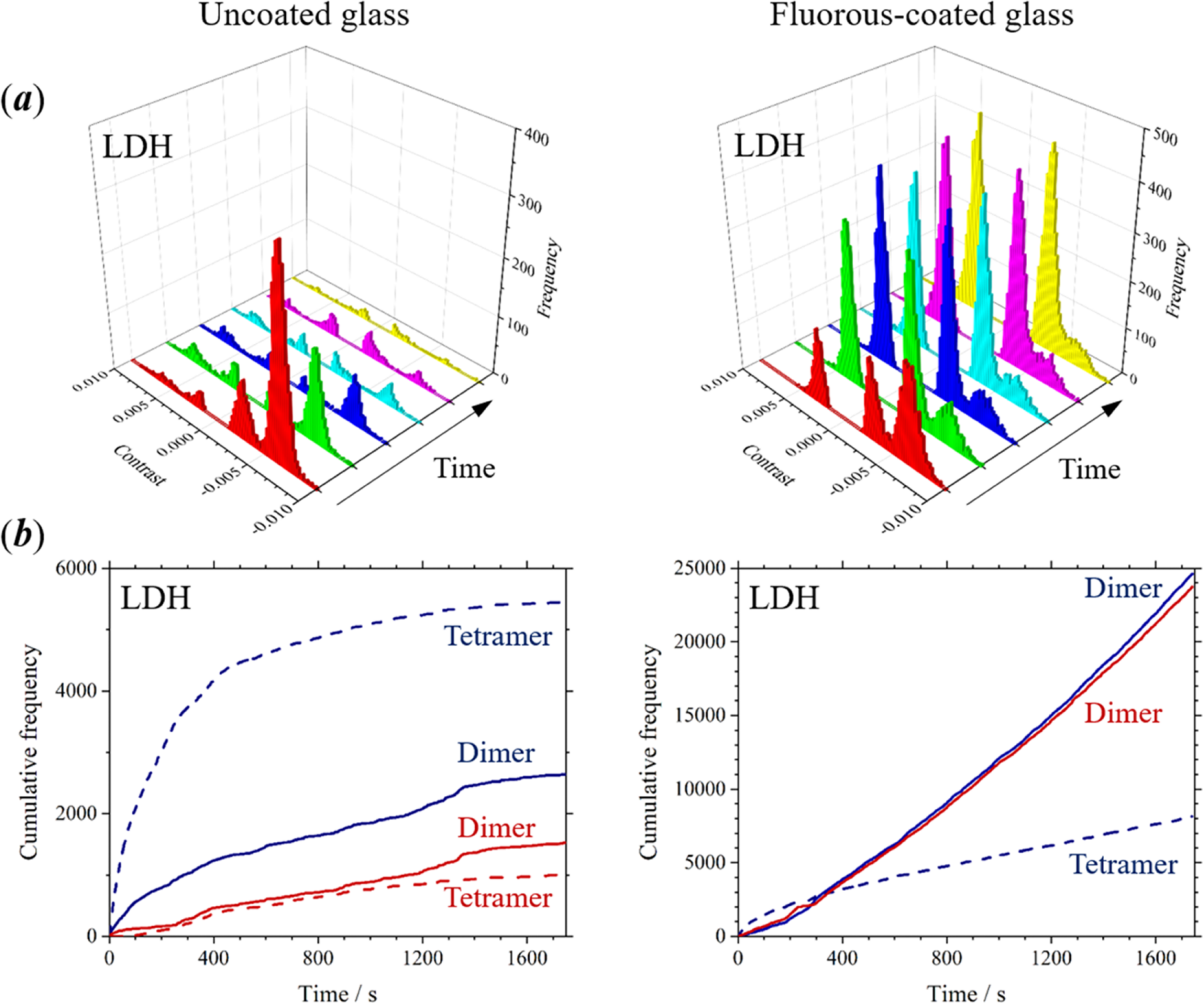
(a) Sequential measurements of the frequency for adsorption of LDH onto uncoated cover glasses (left) and fluorous-coated cover glasses (right). Each of the histograms represents an interval of 5 min (total experiment time was 30 min). Data were obtained after a 10 μL volume of a 20 nM solution of LDH in T50 buffer was dispensed (no further additions of protein were made after the start of the experiment). (b) Cumulative frequency for adsorption (blue) and desorption (red) of LDH on uncoated (left) and fluorous-coated (right) glass (tetramer—dashed line; dimer—solid line).

By performing iSCAT microscopy on different timescales, we have been able to distinguish between monomers, dimers, and tetramers of LDH based on the contrast observed for adsorption and desorption of protein on fluorous surfaces (see the Supporting Information; Figure S5; a trimer was not observed in these experiments). The dissociation of the tetramer of LDH into dimeric protein complexes is kinetically arrested at the experimental concentration of 20 nM. On uncoated glass, there is a high frequency of adsorption events for LDH tetramers and a low frequency for LDH dimers at the start of the experiment; however, these values decline rapidly in subsequent measurements (see Figure 4a, left). The tetramers and dimers are detected as separate peaks at high and low negative contrast, respectively, and the time interval between each histogram shown in Figure 4a is 5 min. The frequencies of desorption events from the tetramer and dimer on uncoated glass (which are detected as corresponding peaks at positive contrast values) are substantially lower than the frequencies for adsorption, with a greater difference observed for the tetramer. On fluorous-coated glass, the frequency of adsorption events for the tetramers and dimers are similar at the start of the experiment; however, the frequency decreases for the tetramer but increases for the dimer in subsequent measurements (see Figure 4a, right). The frequency of desorption events for the dimer is similar to that for adsorption throughout the experiment, but there were no desorption events detected for the tetramer on fluorous-coated surfaces.

The corresponding plots for the cumulative frequency of adsorption and desorption of protein on both types of surfaces are shown in Figure 4b. On uncoated glass (left), the cumulative frequency plots for both adsorption and desorption of dimers and tetramers exhibit a negative curvature and tend toward a shallow gradient at longer times. On fluorous-coated glass (right), the cumulative frequency plots exhibit a positive curvature, between 0 and 400 s, for both adsorption and desorption of the dimer (due to its increasing concentration) and negative curvature over this same interval for adsorption of the tetramer (due to its decreasing concentration). After 400 s, the plots for the cumulative frequency of adsorption and desorption of the dimer, and adsorption of the tetramer, are linear and possess a steep gradient. At this stage, the concentrations of both tetramer and dimer in solution were constant (at equilibrium). It is surprising that desorption events for the tetramer were not observed. However, if adsorption of tetramer was irreversible, then its concentration should be reducing at times >400 s, and the plot of the cumulative frequency for adsorption of the tetramer would show a negative curvature until a zero gradient is reached.

The results shown in Figure 4 suggest that, initially, the dissociation of the tetramer complex is kinetically arrested following the dilution of a sample of LDH to 20 nM, but the perfluoroalkane brush promotes the dissociation of the tetramer into dimer complexes following adsorption on the surface. The analysis of the interferometric images fails to detect desorption events for LDH in which there is simultaneous dissociation of a tetramer into dimer complexes. More than likely, such a desorption event leads to contrast changes in the interferometric images that are spread out across a large number of frames in the video recording. Consequently, this type of desorption event, accompanied by complex dissociation, is not resolved by the analysis software. The positive curvature in the plot of cumulative frequency for the dimer indicates that the concentration of this component is increasing, whereas the negative curvature for the tetramer indicates a lowering of its concentration. The cumulative frequency for both adsorption and desorption of the dimer tends to a linear function toward the end of the experiment. This is the expected behavior for a sample containing a fixed concentration of components, and, in this example, it indicates that a thermodynamic equilibrium has been reached between the tetramer and dimer complexes at the conclusion of the experiment.

In the data shown in Figure 4, there is no clear evidence of the existence of monomers and trimers of LDH. We have been able to observe the presence of monomers of LDH at early times (<60 s) following the addition of a 20 nM solution to a fluorous-coated surface, but the monomer cannot be distinguished from the dimer complexes at later times (see Figure S5).

We could speculate on the molecular mechanism for catalysis of the dissociation of LDH tetramers on a perfluoroalkane brush. The association of LDH with a tetramer is likely to be facilitated by hydrophobic interactions mediated by the amino acid residues that become embedded within the interfacial regions between protein units. By concealing hydrophobic residues on the surface of the protein units, the tetramer has a lower free energy in aqueous solution relative to trimers, dimers, or monomers. The subsequent dilution of a solution to a 20 nM concentration must favor the dissociation of the tetramer. This process will be slow due to hydrophobic regions of the protein being exposed to a polar solvent, and resulting in a high energy barrier to dissociation. We suspect that the transition state for the dissociation of a protein multimer into monomers is stabilized on a perfluoroalkane brush. This is because the exposed hydrophobic residues on the protein monomer could be directed toward the fluorous interface and concealed from the aqueous solvent. The hydrophobic amino acid residues will not have a strong affinity to a perfluoroalkane brush, and, as the protein desorbs from the fluorous interface, it can simultaneously refold into a soluble monomer structure.

The results shown in Figure 4 suggest the lowering of an energy barrier for the dissociation of LDH tetramers into the dimer complexes, with rapid attainment of thermodynamic equilibrium. We have also considered the possibility that the sequential steps of adsorption and desorption of LDH from the fluorous surface lead to denaturing of the protein sample. To investigate this, we have monitored adsorption/desorption events of lactate dehydrogenase (LDH) on a perfluoroalkane brush over a total time interval of 45 min. By extending the observation time in excess of 30 min, the frequency of absorption events of the tetramer starts to increase (these data are shown in Figure S6). Between 30 and 45 min, the volume of the sample droplet on the fluorous-coated surface has been substantially reduced by evaporation of the solvent, which leads to a corresponding increase in the concentration of LDH. Consequently, the position of equilibrium is displaced back from the dimer to the tetramer complex. This observation offers evidence for the absence of denaturation of LDH on fluorous-coated surfaces because the protein is able to re-associate to form a tetramer following an increase in the concentration of the sample.

Ku70/80 is a heterodimeric (mammalian) protein complex of two polypeptides of mass 70 and 80 kDa, respectively, which plays an important role in a DNA repair mechanism by binding to double-strand breaks. The presence of the heterodimer should be detected as a peak at high contrast in frequency distribution measured by iSCAT microscopy, while the presence of either of the monomers, Ku70 or Ku80, should be detected as a single peak at low contrast in the frequency distribution. The difference in mass of the monomers is too small to distinguish between adsorption of the separate units by iSCAT microscopy.

**Figure 5:**
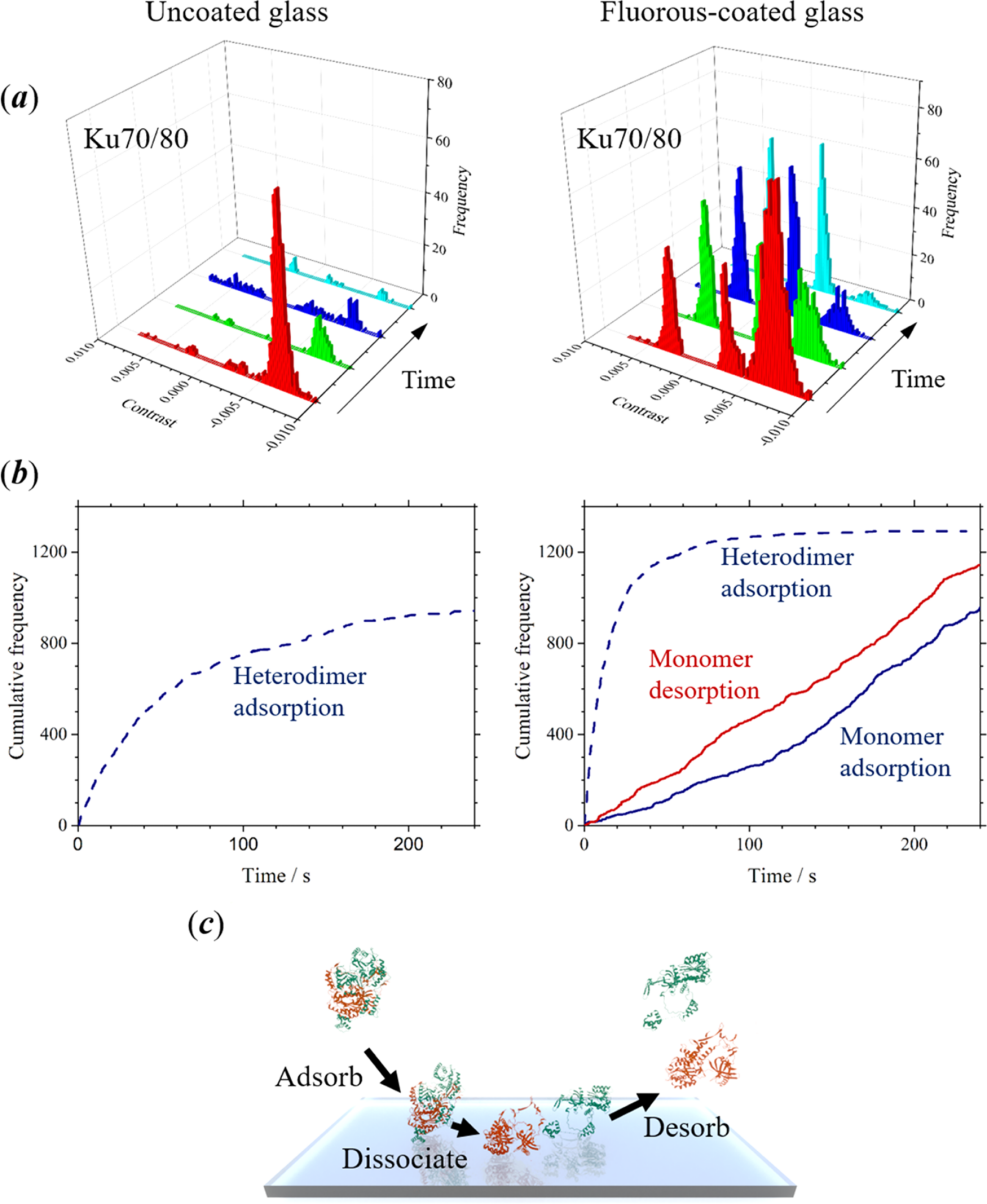
(a) Sequential measurements of the frequency for adsorption of the heterodimer, Ku70/80, and monomer polypeptides, Ku70 and Ku80, onto uncoated cover glasses (left) and fluorous-coated cover glasses (right). Each of the histograms represents an interval of 5 min (total experiment time was 30 min). Data were obtained after a 10 μL volume of a 20 nM solution of Ku70/80 in T50 buffer was dispensed (no further additions of protein were made after the start of the experiment). (b) Cumulative frequency for adsorption (blue) and desorption (red) of Ku70/80 (dashed line) and monomer polypeptides, Ku70 and Ku80 (solid line), on uncoated (left) and fluorous-coated (right) glass. (c) Illustration of a possible mechanism for equilibration of the protein sample, where adsorbed heterodimer dissociates on the fluorous surface, leading to a rapid attainment of thermodynamic equilibrium.

The dissociation of the heterodimer into monomers is also kinetically arrested following dilution of the protein complex to a concentration of 20 nM. On uncoated glass, the adsorption of the protein complex can only be detected for a short interval of time. The interaction between Ku70/80 and uncoated glass appears to be entirely irreversible, and neither the desorption of the heterodimer nor the presence of protein monomers is detected (see Figure 5a, left).

The use of fluorous-coated surfaces enables the equilibration of the heterodimer and monomer units to be observed (see Figure 5a, right). The decrease in the frequency of adsorption indicates that the concentration of the heterodimer declined over the course of the experiment. A peak is not observed in the frequency distribution for the desorption of Ku70/80. Consequently, it appears that the fluorous surface destabilized the heterodimer, leading to dissociation into monomers prior to desorption. As a result of the increasing concentration of monomers following dissociation of the heterodimer, the frequency for both adsorption and desorption of the monomers, Ku70 and Ku80, increase during the course of the experiment.

The cumulative frequency for adsorption of the heterodimer, Ku70/80, and adsorption and desorption of both monomer components is shown in Figure 5b. The presence of the perfluoroalkane brush leads to a thermodynamic equilibrium between monomer subunits and the heterodimer being established at a fast rate. The dilution of the heterodimer to a concentration 20 nM was made at least 10 min prior to the start of the experiment; however, there is still a high concentration of the protein complex observed in the initial interferometric images. Dissociation of the heterodimer is accelerated *via* contact with the fluorous surfaces, and the solution reaches a thermodynamic equilibrium after approximately 100 s. The final composition consists almost entirely of monomer subunits. Unlike the previous example of LDH, the surface-induced dissociation of the heterodimer to monomer subunits, followed by desorption from the perfluoroalkane brush, is apparent in the cumulative frequency plots. Between 0 and 100 s, there is a greater frequency of desorption compared with adsorption of the monomer on the fluorous surfaces, and the plot of cumulative frequency for desorption exhibits a steeper gradient. After 100 s, the frequency of both adsorption and desorption of the dimer are similar, and the plots of cumulative frequency for both adsorption and desorption exhibit a similar gradient. A possible mechanism for the interaction of the heterodimer with a perfluoroalkane brush is shown in Figure 5c.

### Interaction of Lipid Vesicles with a Perfluoroalkane Brush

Large unilamellar vesicles (LUVs; with diameters between 0.1 and 1.0 μm) have been used to encapsulate proteins and nucleic acids for single-molecule studies.^53−55^ In these experiments, the LUVs are tethered to the measurement surface *via* avidin–biotin chemistry, and the encapsulated biomolecule is able to diffuse freely. As a consequence, any deleterious effects on the conformational freedom of the biomolecule caused by direct tethering to measurement surfaces are avoided. Unlike giant unilamellar vesicles (GUVs), LUVs can rupture on uncoated glass surfaces, and passivation is essential in these types of single-molecule experiments.^56^

**Figure 6:**
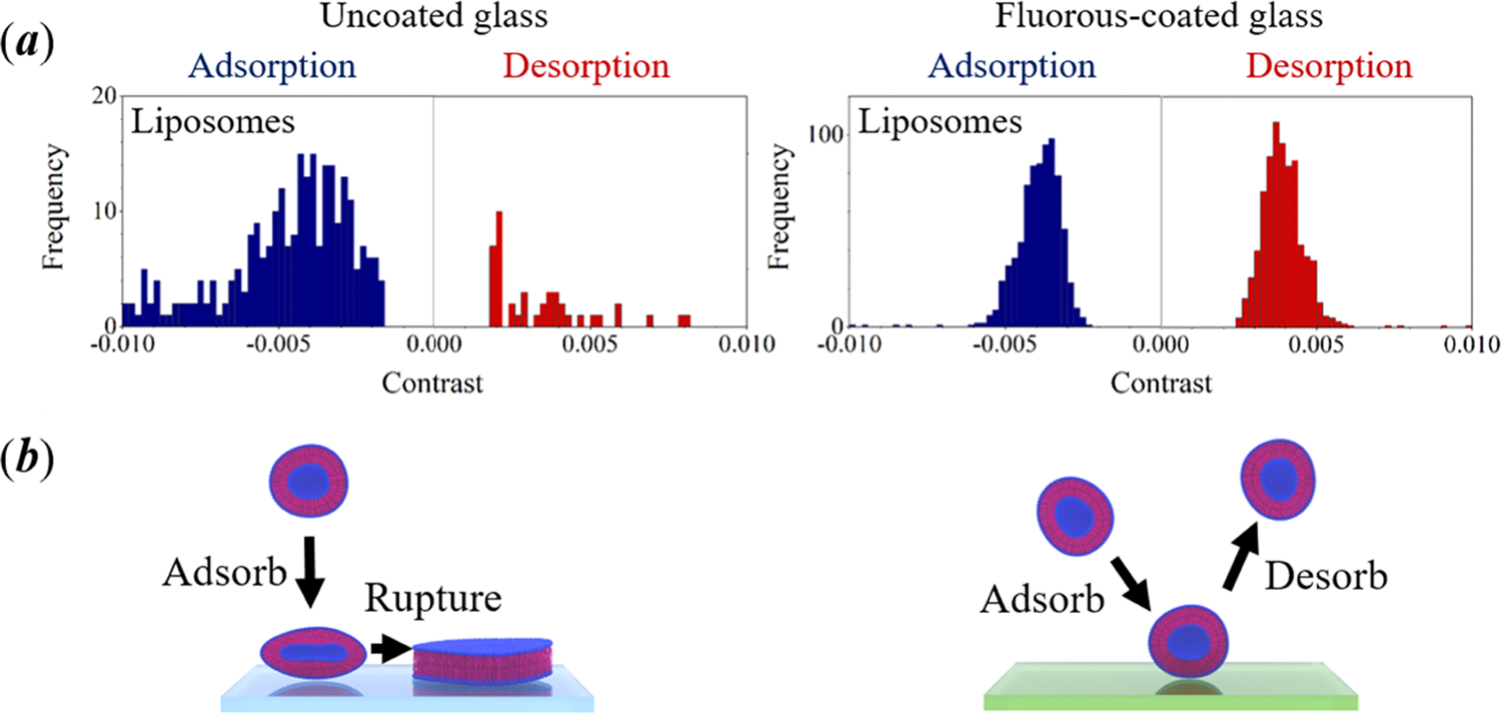
(a) Histograms of the frequency for adsorption (blue) and desorption (red) of 0.1 μm-diameter lipid vesicles onto uncoated cover glasses (left) and fluorous-coated cover glasses (right). The duration of each experiment was 60 s. Data were obtained after a 10 μL volume of a dilute suspension in phosphate-buffered saline was dispensed. The total number of counts for adsorption events relative to desorption events of lipid vesicles are 223/36 (uncoated) and 764/764 (coated). The cumulative counts of adsorption and desorption events as a function of time for lipid vesicles on both types of surface are provided in the Supporting Information. (b) Illustration of a possible mechanism of interaction of lipid vesicles with uncoated (left) and fluorous-coated (right) glass.

Using a monodisperse suspension of LUVs comprising a single component (a saturated phosphocholine lipid), we have observed the frequency of adsorption and desorption on both uncoated glass and fluorous-coated glass; see Figure 6a. The peaks in the frequency distribution for the experiments using uncoated glass (left) are poorly defined and spread across a broad range of contrast values for adsorption (blue) and desorption (red), where the frequency of desorption is much reduced (the total number of desorption events is only 16% of the total number of adsorption events). In this case, the fate of many of the adsorption events is the rupture of the lipid vesicle to form a planar lipid bilayer, a mechanism shown on the left in Figure 6b. In contrast, using fluorous-coated glass (right), there are clearly defined histogram peaks for both adsorption and desorption centered at a contrast of *ca*. ±0.004, where the frequency of adsorption matches that for desorption on the fluorous coating (in this case, the same total of 764 was obtained for adsorption and desorption events). In this case, the adsorption of lipid vesicles is reversible, and the desorption of intact vesicles always takes place immediately or a short time later; a mechanism shown on the right in Figure 6b. The plots for the cumulative frequency for adsorption and desorption of vesicles from the fluorous-coated surface are both approximately linear (with similar gradients; see Figure S7).

## Conclusions

The present work has demonstrated that more information can be extracted using iSCAT microscopy by grafting a perfluoroalkane brush onto the measurement surface. In particular, with this type of surface, quantitative information can be obtained from the detection of both adsorption and desorption of protein, where the latter is often undetected on an uncoated glass surface. The fluorous-coated surfaces resist irreversible adsorption of protein and ensure that the concentration of proteins in dispensed samples remains constant. Typically, biological samples need to be diluted immediately prior to single-molecule experiments, where the desired concentration of target components are somewhere between 10 nM and 10 pM. Dilution will often lead to a significant shift in the position of thermodynamic equilibrium between multimeric and monomeric components, and we have shown that this change in the position of equilibrium can be monitored precisely using fluorous-coated surfaces, which do not perturb the equilibrium further by irreversible adsorption of components. Furthermore, the use of fluorous coatings appears to accelerate the attainment of thermodynamic equilibrium in protein samples, where kinetically arrested states of protein complexes (*i.e.*, LDH, Ku70/80) are destabilized by interaction with the fluorous compounds. Therein lies a more significant reason for the use of fluorous-coated surfaces in single-molecule experiments using iSCAT microscopy, that is, to ensure precise measurement of the components and their interactions in a biological sample. The state-of-the-art method for surface passivation in single-molecule experiments is a coating of poly(ethylene glycol) (PEG). However, this type of passivation is inappropriate for iSCAT microscopy due to the conformational flexibility of PEG and the lack of uniformity of the grafted-polymer coating. Perfluoroakane brushes offer a reliable approach to eliminate the irreversible absorption of molecules for this label-free approach to single-molecule imaging. In the future, it is possible that the fluorous-coated surfaces could be used to direct specific binding of biomolecules for single-molecule imaging. This would require the covalent modification of oligonucleotides or proteins with a short perfluoroalkane tag, which will tether the target molecules *via* the fluorous effect following the insertion of the tag into the interstices in the molecular coating. At this time, it will be possible to compare the perfluoroalkane brush with the state-of-the-art PEG-coated surface for passivating against nonspecific binding in single-molecule fluorescence imaging.

## Materials and Methods

### Preparation of Self-Assembled Monolayers (SAMs) on Glass Substrates

Microscope cover glasses (high precision, 24 × 50 mm^2^, #1.5) made from chemically resistant borosilicate glass D 263 M of the 1st hydrolytic class (Marienfeld) were ultrasonically cleaned in acetone and isopropyl alcohol (IPA) for 5 min and dried under nitrogen. The substrate was subsequently treated with piranha solution (H_2_SO_4_/H_2_O_2_ 7:1) for 10 min, thoroughly rinsed with reverse osmosis (RO) water, and dried under nitrogen before being subjected to oxygen plasma for 10 min at 150 W (Asher RF PlasmaFab Barrel). Gas-phase deposition of a silane-functionalized perfluorinated compound, (heptadecafluoro-1,1,2,2-tetrahydrodecyl) trimethoxysilane (TCI Chemicals), was achieved by placing cover glasses in a Petri dish equidistant from a small vessel containing 200 μL of silane-functionalized perfluoroalkane. The Petri dish was covered with a watch glass and maintained at 60 °C on a hotplate for at least 3 h. The excess silane was removed by sonication in methanol, IPA, and RO water separately for 5 min each. Finally, the slides were baked in an N_2_-flow oven (Carbolite Gero PF 60) at 90 °C overnight.

Adjacent areas of a glass substrate were coated with the hydrocarbon, decyltrimethoxysilane (Fluorochem), and the perfluorinated compound using the same silanization protocol in two sequential steps. The entire surface area of the cover glass was initially coated with the hydrocarbon decyltrimethoxysilane. The photoresist, Microposit S1818, was then spin-coated onto the hydrocarbon surface at 4000 rpm for 30 s and prebaked on a hotplate at 115 °C for 150 s. A chrome mask featuring 50 μm squares was positioned on the photoresist layer and exposed to UV light (365 nm) using a photolithography apparatus (MA6 Mask Aligner, Suss Microtec). The micropattern was transferred to the substrate surface following submersion in a solution containing the developer (MICROPOSIT 351) and water (1:1) for 45 s. The substrate was exposed to an O_2_-plasma barrel (Asher RF PlasmaFab Barrel) at 100 W for 30 s to remove the hydrocarbon coating from the 50 μm squares. The exposed area of the glass surface was subsequently treated with the perfluoroalkane–silane reagent. The remaining resist on the areas above the hydrocarbon coating was stripped in acetone by sonication for 3 min, and the substrate was rinsed thoroughly with IPA and dried with an N_2_ gun.

A Dimension Icon Atomic Force Microscope (Bruker) was used to characterize the surface properties of both fluorous- and alkyl-coated substrates using a OLTESPA-r3 silicon-cantilever tip. The imaging area was 3.85 by 7.7 μm; scan rate was 0.842 Hz, at 512 samples per line across 256 lines. The data shown in Figure 1a are cropped on the left-hand side to a size of 3.85 by c.6.0 μm to place the interface in the center of the image. Contact angle measurements were made using an Easy Drop goniometer (Kruss DSA20E) by dispensing 5 μL of RO water. For the measurement on the uncoated glass slide, the substrate was ultrasonically cleaned in acetone and IPA for 3 min and dried under nitrogen.

### Single-Molecule Experiments

Both uncoated and fluorous-coated cover glasses were cleaned by rinsing in deionized water (×5) and isopropanol (×5), followed by drying under an N_2_ flow. Adsorption and desorption of individual molecules of protein were detected across an imaging area of 10.8 μm by 2.9 μm using a mass photometer (OneMP, Refeyn Ltd.). In these experiments, 10 μL of a 20 nM protein sample in T50 buffer was placed on the cover-glass surface. For uncoated surfaces, a silicon gasket (Refeyn) was used to confine the sample; these were not needed for fluorous-coated surfaces due to the hydrophobic property of the coating [T50 buffer: 10 mM Tris–HCl, pH 8.0, and 50 mM NaCl].

Either individual or sequences of video recordings from interferometric scattering microscopy were obtained for a duration of 60 or 180 s. Single events corresponding to surface adsorption and desorption of protein molecules were identified using AcquireMP software (Refeyn Ltd.). Data analysis was performed using DiscoverMP software (Refeyn Ltd.) and OriginPro 2021 (OriginLab).

The following proteins were used in experiments: BSA (molecular weight, MW, 66 kDa; Sigma-Aldrich, A2153-10G), streptavidin (MW 55 kDa; invitrogen, S11223), heterogeneous nuclear ribonucleoprotein A1 (MW 35 kDa; expressed in *Escherichia coli* and purified), protein A (from *Staphylococcus aureus*, MW 42 kDa, Sigma-Aldrich, P6031), Ku70/80 (MW 150 kDa), and LDH (MW 147 kDa; Merck, 9001-60-9). A further experiment was performed to examine how the uncoated and fluorous-coated surfaces interact with lipid vesicles comprising a single component, 1-palmitoyl-2-oleoyl-glycero-3-phosphocholine (Avanti Polar Lipids, 850457). A suspension of lipid vesicles was prepared by extrusion through a polycarbonate membrane to give a monodisperse size distribution with a mean particle diameter of approximately 100 nm, in accordance with previously published methods.^57^

## Supporting Information Available

Histograms of the frequency for adsorption and desorption of BSA as a function of the interferometric contrast of single-molecule events from four different regions of interest (Figure S1); histograms of the frequency for adsorption and desorption of streptavidin as a function of the interferometric contrast of single-molecule events from four different regions of interest (Figure S2); the cumulative frequency for adsorption and desorption of BSA on fluorous-coated glass over a time period of 17 min (Figure S3); the cumulative frequency for adsorption and desorption of streptavidin, hnRNP A1, and protein A on fluorous-coated glass over an interval of 60 s (Figure S4); sequential measurements of the frequency for adsorption of LDH onto uncoated cover glasses and fluorous-coated cover glasses (Figure S5); bar chart illustrating the frequency of adsorption of LDH dimer and LDH tetramer onto fluorous-coated glass over sequential 3-min intervals of time (Figure S6); the cumulative frequency for adsorption and desorption of liposomes on uncoated and fluorous-coated glass (Figure S7); back-of-the-envelope calculation of the cumulative frequency of protein adsorption on measurement surfaces (Appendix) (PDF)

## Abbreviations

iSCAT: interferometric scattering
TIRFM: total internal reflection fluorescence microscopy
RNA: ribonucleic acid
BSA: bovine serum albumin
PEG: poly(ethylene glycol)
DNA: deoxyribose nucleic acid
SAM: self-assembled monolayer
AFM: atomic force microscopy
LDH: lactate dehydrogenase
IPA: isopropyl alcohol
RO: reverse osmosis
MW: molecular weight
m: monomer
d: dimer
tr: trimer
te: tetramer
