# Supplementary material for: Surface Passivation with a Perfluoroalkane Brush Improves the Precision of Single-Molecule Measurements
Source: ACS Appl Mater Interfaces. 2022 Oct 28;14(44):49604–16. doi: 10.1021/acsami.2c16647 (PMC9650645; doi:10.1021/acsami.2c16647)
Supplement: Supplementary file 1 — am2c16647_si_001.pdf [file am2c16647_si_001.pdf]

SUPPORTING INFORMATION

Surface passivation with a perfluoroalkane brush improves the precision of single-molecule measurements

Carlos J. Bueno-Alejo[1,2]‡, Marina Santana Vega[3]‡, Amanda K. Chaplin[2,4], Chloe Farrow[1,2], Alexander Axer[5], Glenn A. Burley[5], Cyril Dominguez[2,4], Hesna Kara[2,4], Vasileios Paschalis[2,4], Sumera Tubasum[2,4], Ian C. Eperon[2,4], Alasdair W. Clark[3] and Andrew J. Hudson[1,2]**

[1]School of Chemistry, University of Leicester, University Road, Leicester, LE1 7RH. United Kingdom.

[2]Leicester Institute of Structural & Chemical Biology, Henry Wellcome Building, University of Leicester, Lancaster Road, LE1 7HB. United Kingdom.

[3]School of Engineering, Advanced Research Centre, University of Glasgow, 11 Chapel Lane, Glasgow, G11 6EW. United Kingdom.

[4]Department of Molecular and Cellular Biology, Henry Wellcome Building, University of Leicester, Lancaster Road, LE1 7HB. United Kingdom.

[5]Strathclyde Centre for Molecular Bioscience & Department of Pure & Applied Chemistry, University of Strathclyde, 295 Cathedral Street, Glasgow, G1 1XL. United Kingdom.

Corresponding Author:

* Alasdair W. Clark (ORCID 0000-0002-1643-8694) and Andrew J. Hudson (ORCID 0000-0003-1849-9666).

alasdair.clark@glasgow.ac.uk; andrew.hudson@leicester.ac.uk.

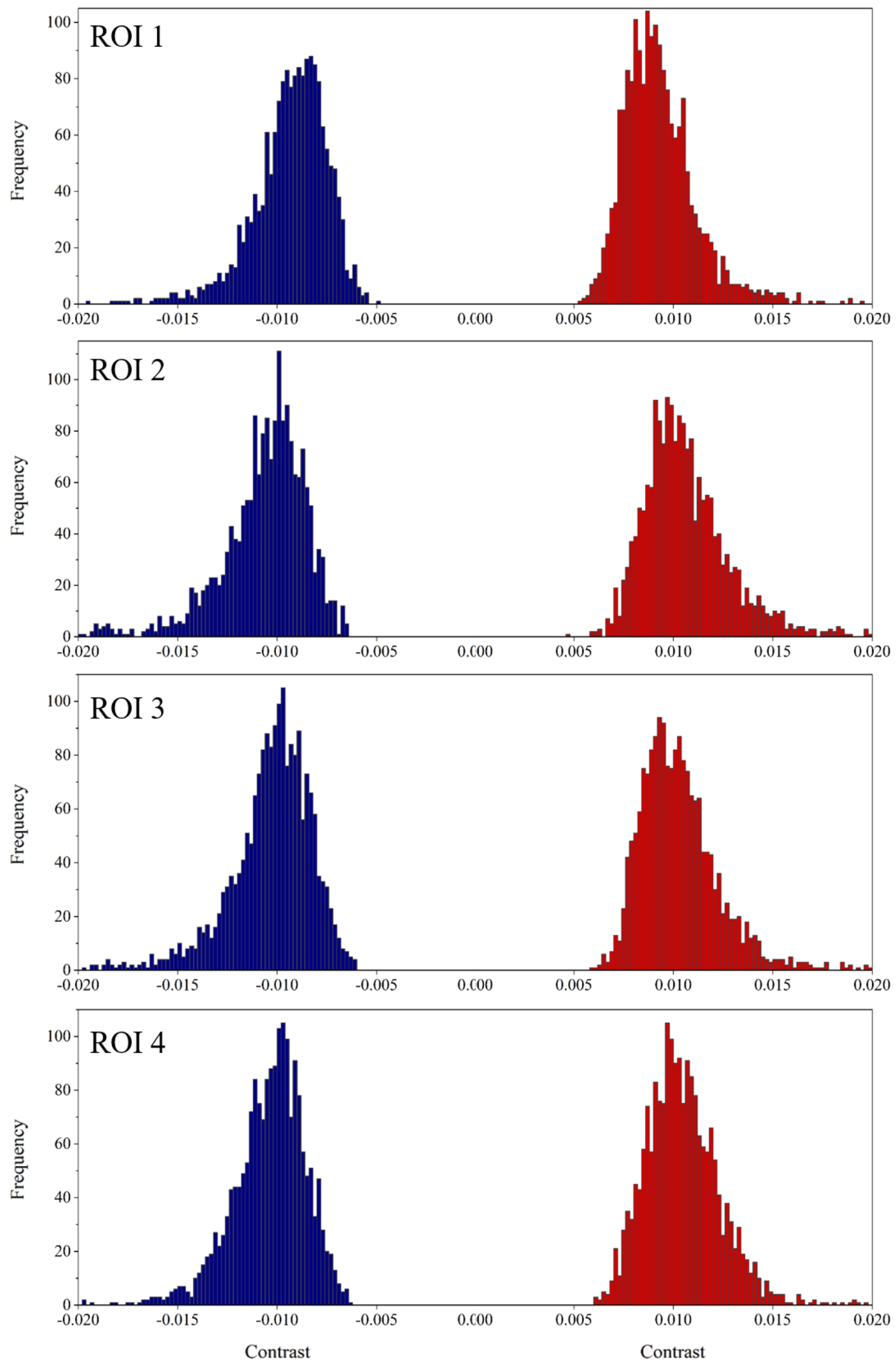

Figure S1. Histograms of the frequency for adsorption (blue) and desorption (red) of BSA as a function of the interferometric contrast of single-molecule events. A 20 µl volume of a 20 nM solution of BSA in T50 buffer was dispensed onto a fluorous-coated cover glass. Data was obtained across 60 s from each of four different regions of interest (ROIs).

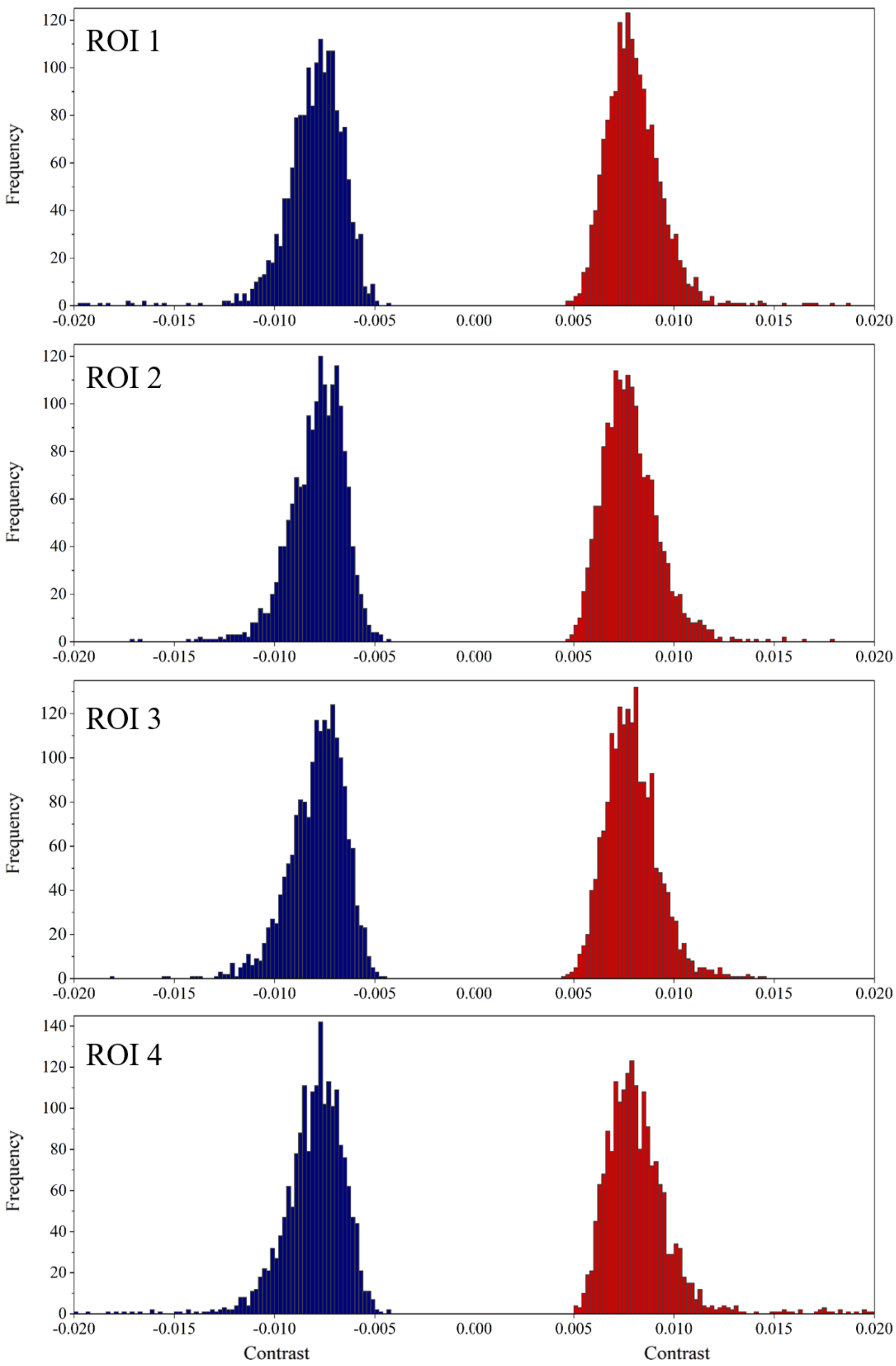

Figure S2. Histograms of the frequency for adsorption (blue) and desorption (red) of Straptavidin as a function of the interferometric contrast of single-molecule events. A 20 μl volume of a 20 nM solution of Streptavidin in T50 buffer was dispensed onto a fluorous-coated cover glass. Data was obtained across 60 s from each of four different regions of interest (ROIs).

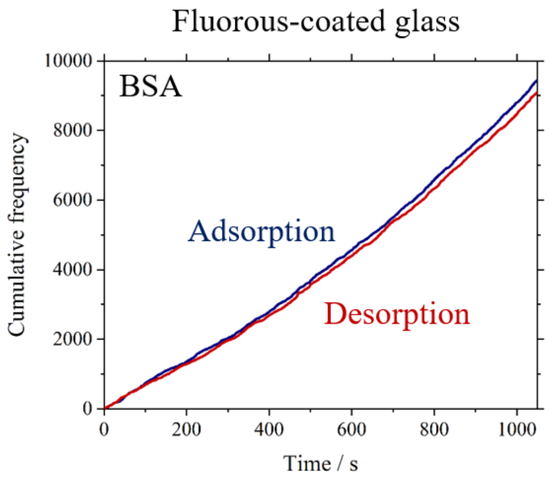

Figure S3. The cumulative frequency for adsorption (blue) and desorption (red) of BSA on fluorous-coated glass.

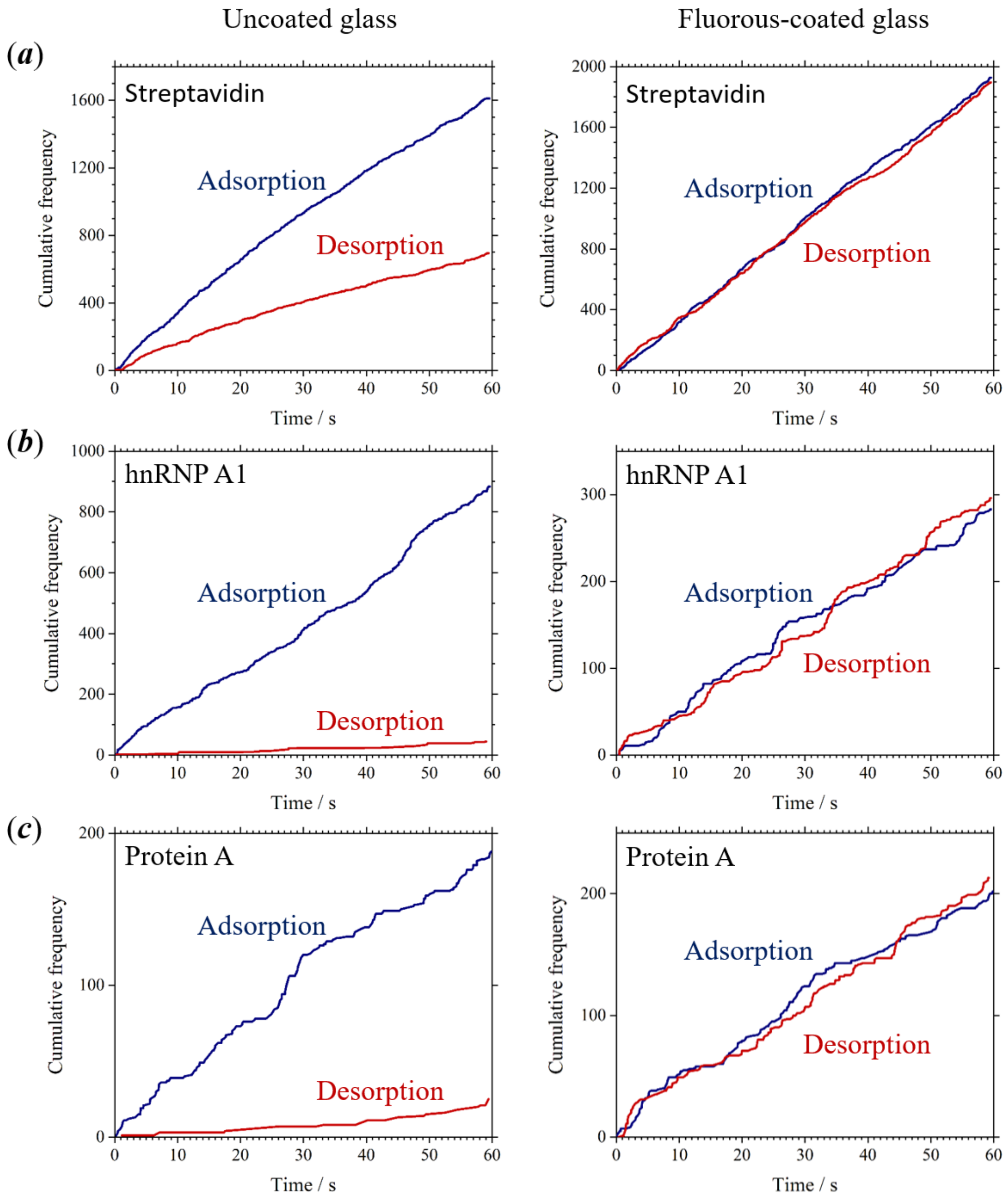

Figure S4. The cumulative frequency for adsorption (blue) and desorption (red) of ***(a)*** streptavidin, ***(b)*** hnRNP A1 and ***(c)*** protein A on fluorous-coated glass over an interval of 60 seconds.

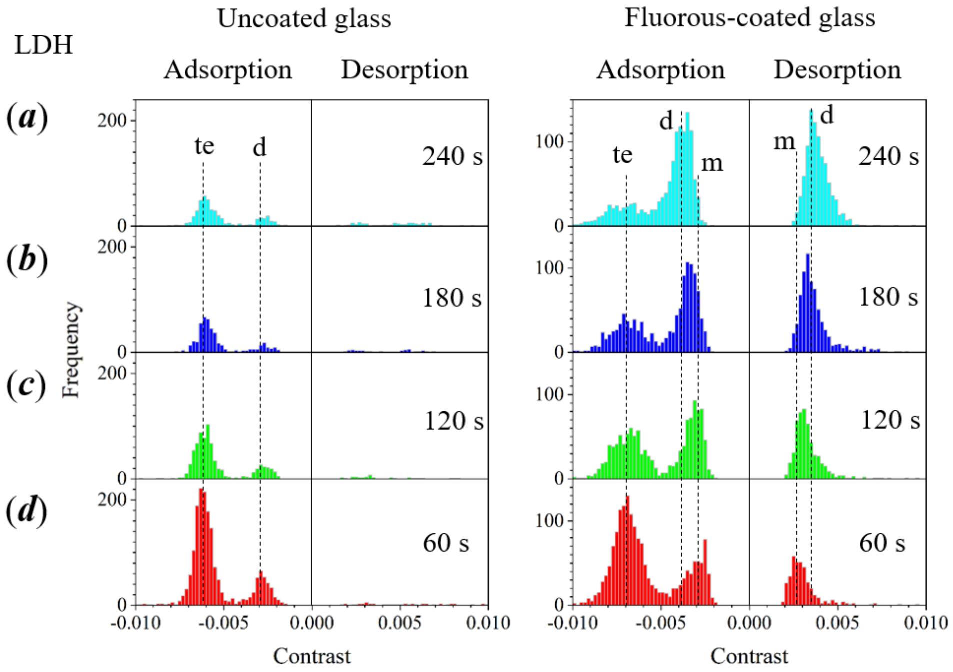

Figure S5. Sequential measurements of the frequency for adsorption of LDH onto uncoated cover glasses (left) and fluorous-coated cover glasses (right). iSCAT measurements were made in the following time intervals: ***(a)*** 0 – 60 s; ***(b)*** 60 – 120 s; ***(c)*** 120 – 180 s; ***(d)*** 180 – 240 s. Each of the histograms represents an interval of 60 seconds (total experiment time shown in the figure is 5 minutes). Difference contrast values can be associated with the monomer (m), dimer (d) and tetramer (te). Data was obtained after a 10 μl volume of a 20 nM solution of LDH in T50 buffer was dispensed (no further additions of protein were made after the start of the experiment).

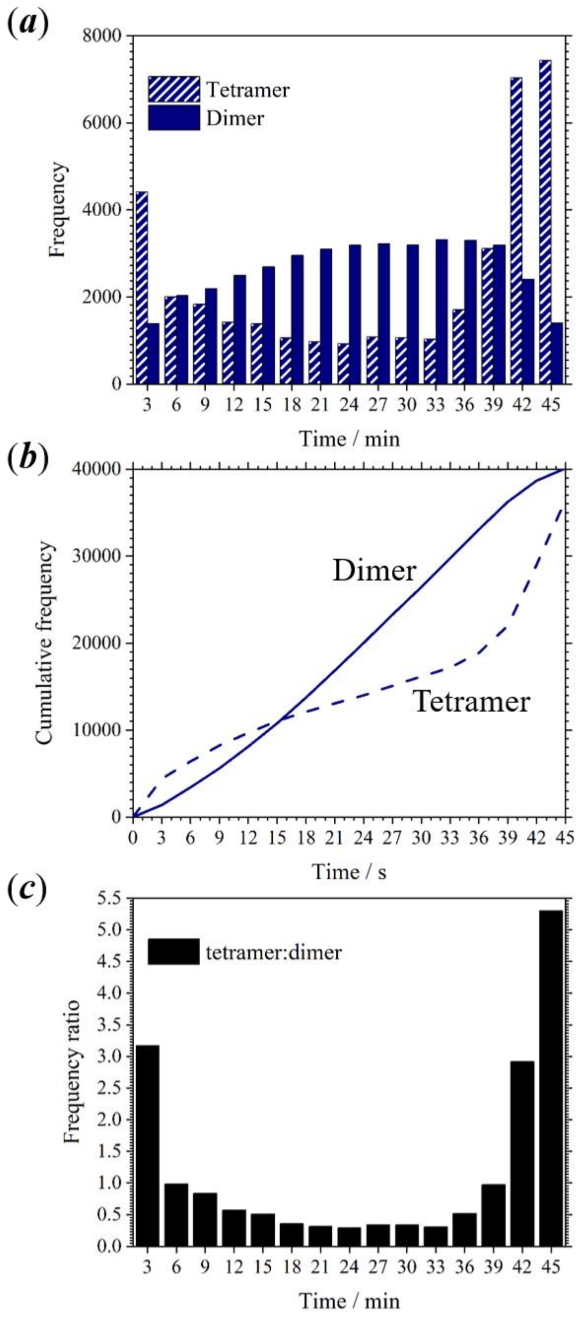

Figure S6. (*a*) Bar chart illustrating the frequency of adsorption of LDH dimer (solid) and LDH tetramer (diagonal line pattern) onto fluorous-coated glass. The data was acquired for 45 minutes and partitioned into 3 minute intervals of time. A 20 μl volume of a 20 nM solution of LDH in T50 buffer was dispensed onto the cover glass, and the droplet evaporated gradually during the experiment. **(*b*)** The cumulative frequency for adsorption of LDH dimer (solid) and LDH tetramers (dashed) onto fluorous-coated glass. **(*c*)** The frequency ratio for adsorption of the tetramer to the dimer of LDH onto fluorous-coated glass.

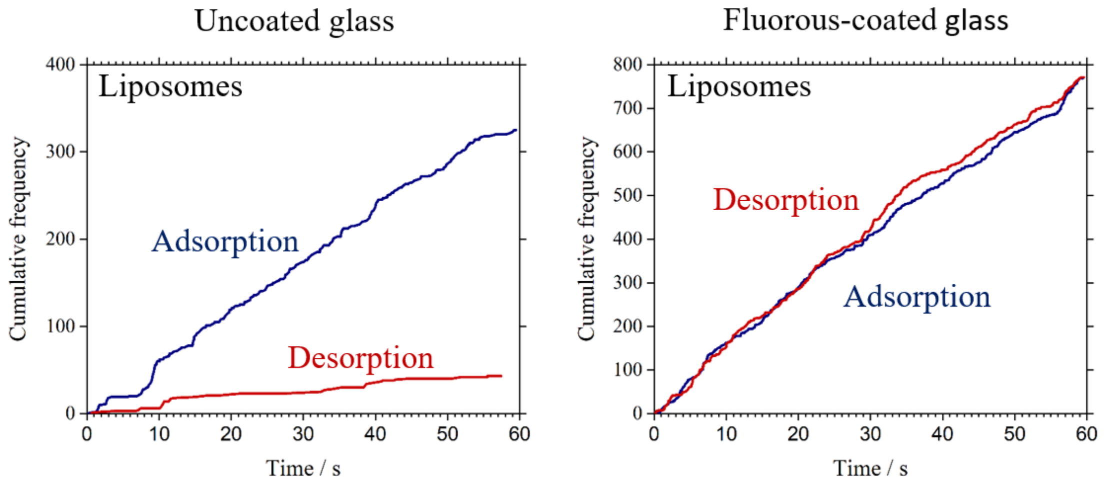

Figure S7. The cumulative frequency for adsorption and desorption of liposomes on uncoated and fluorous-coated glass.

Appendix: Back-of-the-envelope calculation of the cumulative frequency of protein adsorption on measurement surfaces

A simplified model was adopted for a sessile drop corresponding to the protein sample dispensed onto the measurement surface: a cylindrical droplet shape. For the 20 μl sample volume, the cylinder was assigned a 4 mm diameter and a 1.6 mm height.

The protein sample of 20 nM concentration (*i.e.* 2.4×10^{11} molecules in 20 μl) was initially confined to this volume. We considered the axial diffusion of protein molecules only over a time interval, t = 5 minutes. The one-dimensional displacement of protein would follow a normal distribution with a standard deviation of $(2\ D\ t)^{1/2}$; where the diffusion coefficient of protein, D is ca.10^{-6} cm^2 s^{-1}.

In the model, the diffusion of protein was not confined to the volume of the cylindrical droplet shape on the measurement surface. A value was obtained for the number of protein molecules that would have a final location below the measurement surface. Note that this fraction would represent a lower limit to the collision frequency of protein with the cover glass, since many more protein trajectories will involve a collision with the cover glass but remain within the original droplet volume after 5 minutes.
